# High familial burden of cancer correlates with improved outcome from immunotherapy in patients with NSCLC independent of somatic DNA damage response gene status

**DOI:** 10.1186/s13045-022-01226-2

**Published:** 2022-01-21

**Authors:** Alessio Cortellini, Raffaele Giusti, Marco Filetti, Fabrizio Citarella, Vincenzo Adamo, Daniele Santini, Sebastiano Buti, Olga Nigro, Luca Cantini, Massimo Di Maio, Joachim G. J. V. Aerts, Emilio Bria, Federica Bertolini, Miriam Grazia Ferrara, Michele Ghidini, Francesco Grossi, Annalisa Guida, Rossana Berardi, Alessandro Morabito, Carlo Genova, Francesca Mazzoni, Lorenzo Antonuzzo, Alain Gelibter, Paolo Marchetti, Rita Chiari, Marianna Macerelli, Francesca Rastelli, Luigi Della Gravara, Stefania Gori, Alessandro Tuzi, Michele De Tursi, Pietro Di Marino, Giovanni Mansueto, Federica Pecci, Federica Zoratto, Serena Ricciardi, Maria Rita Migliorino, Francesco Passiglia, Giulio Metro, Gian Paolo Spinelli, Giuseppe L. Banna, Alex Friedlaender, Alfredo Addeo, Corrado Ficorella, Giampiero Porzio, Marcello Tiseo, Marco Russano, Alessandro Russo, David James Pinato

**Affiliations:** 1 Department of Biotechnology and Applied Clinical Sciences, University of L’Aquila, L’Aquila, Italy; 2 Division of Cancer, Department of Surgery and Cancer, ICTEM Building, Hammersmith Hospital, Imperial College London, Du Cane Road, London, W12 0HS UK; 3 Medical Oncolgy, St. Andrea Hospital, Rome, Italy; 4 Medical Oncology, Campus Bio-Medico University, Rome, Italy; 5 Medical Oncology, A.O. Papardo and Department of Human Pathology, University of Messina, Messina, Italy; 6 Medical Oncology Unit, University Hospital of Parma, Parma, Italy; 7 Medical Oncology, ASST-Sette Laghi, Varese, Italy; 8 Department of Pulmonary Diseases, Erasmus Medical Center, Rotterdam, The Netherlands; 9 Oncology Clinic, Università Politecnica Delle Marche, Ospedali Riuniti Di Ancona, Ancona, Italy; 10 Department of Oncology, University of Turin and Medical Oncology, AO Ordine Mauriziano, Turin, Italy; 11 Comprehensive Cancer Center, Fondazione Policlinico Universitario “A. Gemelli” IRCCS, Rome, Italy; 12 Department of Translational Medicine and Surgery, Università Cattolica del Sacro Cuore, Romae, Lazio Italy; 13 Dipartimeto Di Oncologia Ed Ematologia, AOU Policlinico Modena, Modena, Italy; 14 Medical Oncology Unit, Fondazione IRCCS Ca’ Granda Ospedale Maggiore Policlinico, Milan, Italy; 15 Division of Medical Oncology, University of Insubria, Varese, Italy; 16 Struttura Complessa Di Oncologia Medica E Traslazionale, Azienda Ospedaliera Santa Maria Di Terni, Terni, Italy; 17 Thoracic Medical Oncology, Istituto Nazionale Tumori ‘Fondazione G Pascale’, IRCCS, Napoli, Italy; 18 UOC Clinica Di Oncologia Medica, IRCCS Ospedale Policlinico San Martino, Genoa, Italy; 19 Dipartimento Di Medicina Interna E Specialità Mediche, Università Degli Studi Di Genova, Genoa, Italy; 20 Department of Oncology, Careggi University Hospital, Florence, Italy; 21 Medical Oncology (B), Policlinico Umberto I, “Sapienza” University of Rome, Rome, Italy; 22 Department of Clinical and Molecular Medicine, Sapienza University, Rome, Italy; 23 Medical Oncology, Ospedali Riuniti Padova Sud “Madre Teresa Di Calcutta”, Monselice, Italy; 24 Department of Oncology, University Hospital Santa Maria Della Misericordia, Udine, Italy; 25 Medical Oncology, Fermo Area Vasta 4, Fermo, Italy; 26 Pneumo-Oncology Unit, Monaldi Hospital, Naples, Italy; 27 Oncology Unit, IRCCS Ospedale Sacro Cuore Don Calabria, Negrar, VR Italy; 28 Dipartimento Di Terapie Innovative in Medicina E Odontoiatria, Università G. D’Annunzio, Chieti-Pescara, Chieti, Italy; 29 Clinical Oncology Unit, S.S. Annunziata Hospital, Chieti, Italy; 30 Medical Oncology, F. Spaziani Hospital, Frosinone, Italy; 31 Medical Oncology, Santa Maria Goretti Hospital, Latina, Italy; 32 Pneumo-Oncology Unit, St. Camillo-Forlanini Hospital, Rome, Italy; 33 Department of Oncology, University of Turin, San Luigi Hospital, Orbassano, TO Italy; 34 Department of Medical Oncology, Santa Maria Della Misericordia Hospital, Azienda Ospedaliera Di Perugia, Perugia, Italy; 35 UOC Territorial Oncology, AUSL Latina – CdS Aprilia, Aprilia, Italy; 36 Candiolo Cancer Institute, FPO-IRCCS, Candiolo, Turin, Italy; 37 Oncology Department, University Hospital of Geneva, Geneva, Switzerland; 38 Department of Medicine and Surgery, University of Parma, Parma, Italy; 39 Department of Translational Medicine, Università del Piemonte Orientale “A. Avogadro”, Novara, Italy

**Keywords:** Family history of cancer, DDR genes, NSCLC, Pembrolizumab, Immune checkpoint inhibitors, Immunotherapy

## Abstract

Family history of cancer (FHC) is a hallmark of cancer risk and an independent predictor of outcome, albeit with uncertain biologic foundations. We previously showed that FHC-high patients experienced prolonged overall (OS) and progression-free survival (PFS) following PD-1/PD-L1 checkpoint inhibitors. To validate our findings in patients with NSCLC, we evaluated two multicenter cohorts of patients with metastatic NSCLC receiving either first-line pembrolizumab or chemotherapy. From each cohort, 607 patients were randomly case–control matched accounting for FHC, age, performance status, and disease burden. Compared to FHC-low/negative, FHC-high patients experienced longer OS (HR 0.67 [95% CI 0.46–0.95], *p* = 0.0281), PFS (HR 0.65 [95% CI 0.48–0.89]; *p* = 0.0074) and higher disease control rates (DCR, 86.4% vs 67.5%, *p* = 0.0096), within the pembrolizumab cohort. No significant associations were found between FHC and OS/PFS/DCR within the chemotherapy cohort. We explored the association between FHC and somatic DNA damage response (DDR) gene alterations as underlying mechanism to our findings in a parallel cohort of 118 NSCLC, 16.9% of whom were FHC-high. The prevalence of ≥ 1 somatic DDR gene mutation was 20% and 24.5% (*p* = 0.6684) in FHC-high vs. FHC-low/negative, with no differences in tumor mutational burden (6.0 vs. 7.6 Mut/Mb, *p* = 0.6018) and tumor cell PD-L1 expression. FHC-high status identifies NSCLC patients with improved outcomes from pembrolizumab but not chemotherapy, independent of somatic DDR gene status. Prospective studies evaluating FHC alongside germline genetic testing are warranted.

**Supplementary Information:**

The online version contains supplementary material available at 10.1186/s13045-022-01226-2.

## To the editor,

Pathogenic germline mutations affecting the DNA damage response and repair (DDR) genes are among the few underlying known mechanisms of inherited cancer susceptibility. However, clear inheritable defects like those leading to Lynch syndrome (LS) [1] and hereditary breast-ovarian cancer syndrome (HBOC) [2], explain only a limited part of the family history of cancer (FHC) usually seen in clinic. Acknowledging the immune-sensitive phenotype of cancers related to DDR genes defects [3], we postulated that FHC may be linked to immunotherapy efficacy and demonstrated that a high burden of FHC (FHC-high) is an independent, tumour-agnostic predictor of prolonged overall survival (OS) and progression-free survival (PFS) in a large cohort treated with PD-1/PD-L1 checkpoint inhibitors [4], a finding that led us to hypothesize that the underlying mechanism may relate to pathogenetic DDR genes alterations. To investigate whether FHC correlates with outcomes from immunotherapy in non-small cell lung cancer (NSCLC), we designed this study including two large, matched cohorts of patients with metastatic NSCLC treated with either first-line pembrolizumab (PD-L1 tumor expression ≥ 50%) or chemotherapy [5–9].

**Fig. 1:**
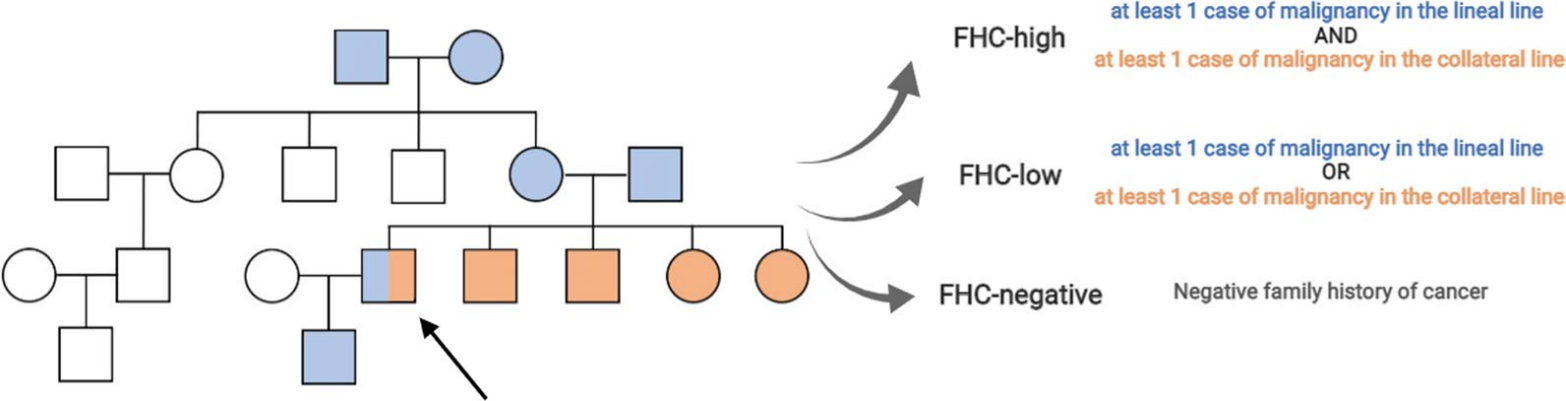
Family history data collection. All oncological disease with malignant potential, both hematological and solid, were screened. Lineal line (descendants or ascendants) and collateral line (non-descentants/ascendants e.g., brothers/sisters) were screened till the second degree (grandparents for lineal line and brothers/sisters for the collateral line). Patients were categorized as follow: FHC-high (in case of at least one cancer diagnosis in both lineal and collateral family lines), FHC-low (in case of at least one cancer diagnosis in either the lineal or collateral line) and FHC-negative (Fig. 1). On the basis of our previous findings (Ref. [4]), FHC-high was considered the group of interest for all analyses

Detailed study methodology is provided as supplementary material. Overall, 167/890 (18.7%) and 88/740 (11.9%) patients were excluded from the pembrolizumab and chemotherapy cohorts, due to missing FHC data, resulting in 723 and 652 patients, respectively. FHC data was collected as previously described and patients were categorized as FHC-high and FHC-low/negative (Fig. 1). Patients’ characteristics are summarized in Additional file 1: Table S1. None of the baseline characteristics were significantly associated with FHC categories in either the pembrolizumab or the chemotherapy cohorts (Additional file 1: Table S2). Additional file 1: Table S3 and Fig. S1 provide detailed FHC information for the 49 FHC-high patients from the pembrolizumab cohort. Lung cancer was the most frequently reported malignancy, without specific family clusters. Cases/controls were randomly paired on the basis of the FHC, age, ECOG-PS, and burden of disease, and 607 patients from the pembrolizumab and chemotherapy cohorts were perfectly paired.

**Fig. 2:**
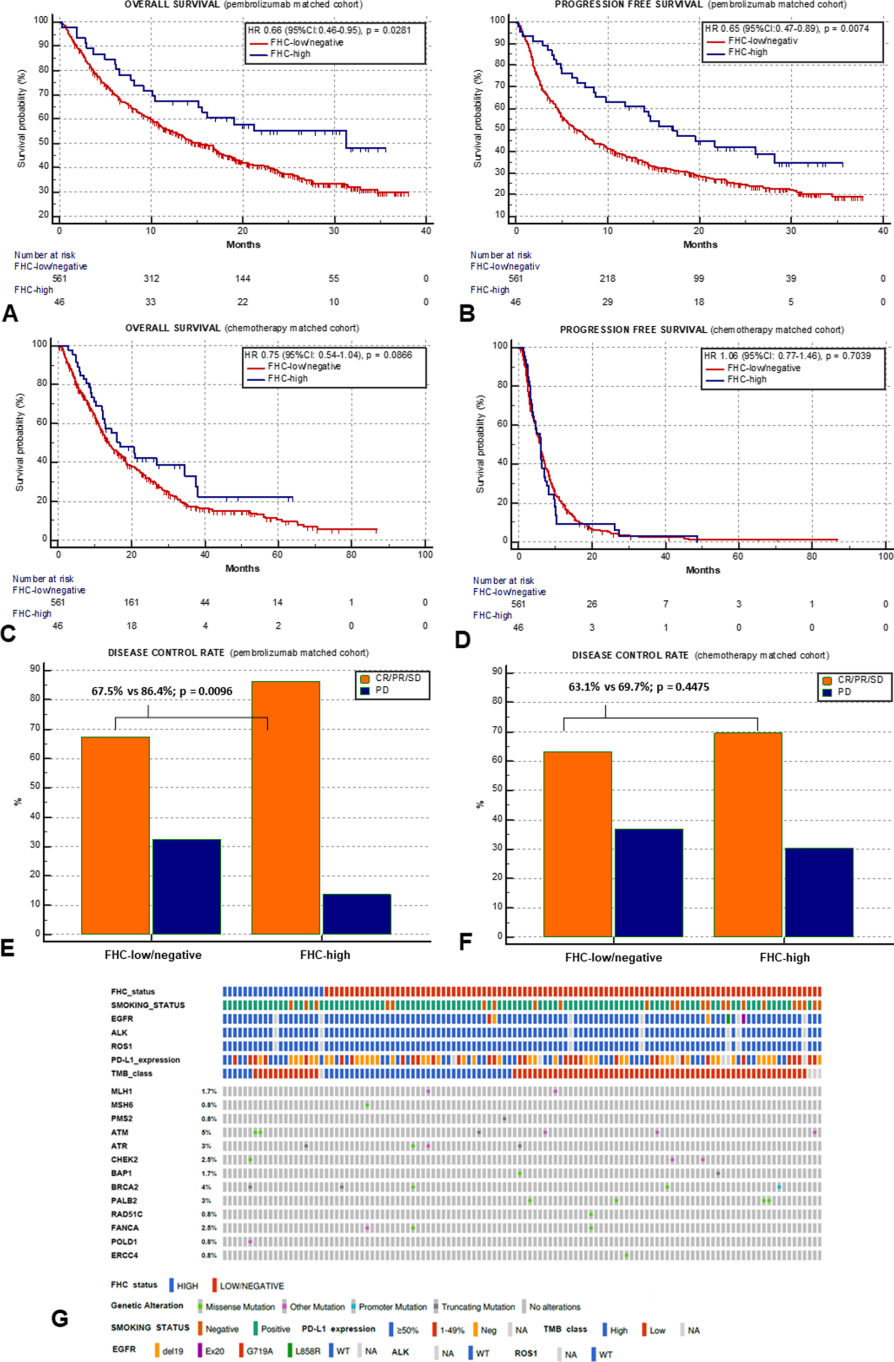
Clinical outcomes analysis according to the FHC across the pembrolizumab and chemotherapy matched cohorts. Median OS and PFS of the entire pembrolizumab cohort were 15.4 months (95% CI 12.8–17.3; 421 events) and 6.9 months (95% CI 5.8–7.9; 523 events), respectively, whilst for the chemotherapy cohort were 14.4 months (95% CI 12.9–16.6; 466 events) and 5.9 months (95% CI 5.3–6.3; 594 events), respectively. The median follow-up was 23.3 months (95% CI 21.8–38.0) for the pembrolizumab cohort and 38.4 months (95% CI 33.1–86.7) for the chemotherapy cohort. Kaplan–Meier survival estimates for OS; pembrolizumab cohort (**A**) FHC-high: 31.3 months (95% CI 15.2–31.3, 21 events) vs FHC-low/negative: 15.3 months (95% CI 12.8–17.5, 327 events), *p* = 0.0281; chemotherapy cohort (**C**) FHC-high: 16.9 months (95% CI 12.1–34.5, 29 events) vs FHC-low/negative: 13.8 months (95% CI 12.3–15.8, 408 events), *p* = 0.0866. Kaplan–Meier survival estimates for PFS; pembrolizumab cohort (**B**) FHC-high: 17.2 months (95% CI 8.6–28.1, 28 events) vs FHC-low/negative: 6.5 months (95% CI 5.4–28.3, 405 events), *p* = 0.0074; chemotherapy cohort (**D**) FHC-high: 5.9 months (95% CI 3.9–6.9, 44 events) vs FHC-low/negative: 5.0 months (95% CI 5.3–6.4, 5090 events), *p* = 0.7039. DCR; pembrolizumab cohort (**E**) FHC-high: 86.4% (95% CI 61.1–118.5) vs FHC-low/negative: 67.5% (95% CI 60.5–75.1), *p* = 0.0096; chemotherapy cohort (**F**) FHC-high: 69.7% (95% CI 44.1–104.5) vs FHC-low/negative: 63.1% (95% CI 56.0–70.1), *p* = 0.4475. (**G**) OncoPrint plot summarizing relevant baseline clinic-pathologic characteristics and the DDR genes profile of the parallel cohort. Patients are clustered according to the FHC status (first row) and in the upper section the smoking status, common actionable biomarkers (including EGFR, ALK and ROS-1), the PD-L1 tumour expression and the TMB category (with a cut off of ≥ vs < 10 mutations/megabase) are reported. The mutational status and its prevalence of selected DDR genes is reported with different colours according to the mutation’s type. Made with cBioPortal oncoprinter, available at: https://www.cbioportal.org/oncoprinter. FHC, family history of cancer; OS, overall survival; PFS, progression free survival; DCR, disease control rate

As compared to FHC-low/negative patients, FHC high achieved a significantly longer OS (31.3 vs. 15.3 months; HR = 0.67 [95% CI 0.46–0.95], *p* = 0.0281; Fig. 2A), and PFS (17.2 vs. 6.5 months; HR = 0.65 [95% CI 0.48–0.89]; *p* = 0.0074; Fig. 2B) and a higher disease control rate (DCR) (86.4% vs 67.5%, *p* = 0.0096; Fig. 2E), within the pembrolizumab cohort. On the contrary, no significant associations were found between FHC and OS (16.9 vs 13.8 months; *p* = 0.0866; Fig. 2C), PFS (5.9 vs. 5.0 months; *p* = 0.7039; Fig. 2D), DCR (69.7% vs. 63.1%; *p* = 0.4475; Fig. 2F) within the chemotherapy cohort . Additional file 1: Table S4 and Fig. S2A–F summarize all the univariable analyses according to the FHC across the entire pembrolizumab and chemotherapy cohorts. The pooled multivariable analysis including both cohorts, with and without the interaction term between the FHC and therapeutic modality (immunotherapy vs chemotherapy) is reported in Additional file 1: Table S5. Of note, a statistically significant interaction was found (*p* = 0.0170) with respect to PFS, highlighting a differential effect of FHC depending on treatment modality.

We used a parallel cohort of 118 patients with NSCLC (20 FHC-high, 16.9% and 98 FHC-low/negative, 83.1%) to explore the implication of somatic DDR gene alterations in explaining FHC-driven benefit. Using the FoundationOne CDx assay we focused on 24 genes among the 324 detectable cancer-related alterations derived from a reference panel defined by Ricciuti et al. [10]. The prevalence of ≥ 1 DDR gene mutation was 20% (4/20) and 24.5% (24/74) for FHC-low/negative and FHC-high patients (*p* = 0.6684). Baseline characteristics and DDR gene profiles are summarized in Fig. 2G. No association between FHC and tumor mutational burden/PD-L1 expression was found (Additional file 1: Fig. S3A-B).

This study identifies FHC-high patients as a subgroup characterised by increased benefit to pembrolizumab, strengthening the putative role FHC as a predictive correlate of benefit following PD-1 inhibition. To elucidate the underlying mechanisms, we focused on somatic DDR defects, since in NSCLC they have been already established as independent predictors of response/survival to PD-1/PD-L1 inhibitors [10], whereas the FHC has found a partial role only in the context of early detection/screening [11, 12], and has never been comprehensively evaluated in advanced patients, probably because of no solid linkage to hereditary syndromes [13, 14]. However, the distribution of DDR defects was not enriched in FHC-high patients, highlighting the complexity of the mechanisms involved, that may go beyond single-hit germline tumour-suppressor genes mutations, as we are used to see in HBOC and LS.

Of note, we did not have PD-L1 expression data for the chemotherapy group, and we were not able to match the clinical cohorts according to the PD-L1 tumor expression. However, we did not find any association between the FHC and the PD-L1 status in either the pembrolizumab or the parallel exploratory cohorts, but we can assume that only 30% of the chemotherapy recipients had a high PD-L1 status [15]. Although our study acknowledges several limitations mainly coming from its design and risk of recalling bias, we provided informative evidence in the context of first-line immunotherapy of NSCLC, confirming that FHC-high patients achieve better outcomes to single-agent pembrolizumab, a finding that requires prospective studies incorporating germline and somatic mutational screening in immunotherapy recipients.

## Supplementary Information

**Additional file 1**. Supplementary materials.

## Data Availability

The datasets used during the present study are available from the corresponding author upon reasonable request.

## Abbreviations

DDR: DNA damage response and repair
LS: Lynch syndrome
HBOC: Hereditary breast-ovarian cancer syndrome
FHC: Family history of cancer
OS: Overall survival
PFS: Progression-free survival
PD-1: Programmed death-1
PD-L1: Programmed death-ligand 1
NSCLC: Non-small cell lung cancer
HR: Hazard ratio
DCR: Disease control rate
