# Supplementary material for: High familial burden of cancer correlates with improved outcome from immunotherapy in patients with NSCLC independent of somatic DNA damage response gene status
Source: J Hematol Oncol. 2022 Jan 21;15:9. doi: 10.1186/s13045-022-01226-2 (PMC8780322; doi:10.1186/s13045-022-01226-2)
Supplement: Supplementary file 1 — Additional file 1. Supplementary materials. [file 13045_2022_1226_MOESM1_ESM.pdf]

High familial burden of cancer correlates with improved outcome from immunotherapy in patients with NSCLC independent of somatic DNA damage response gene status.

By Cortellini A, et al.

Supplementary Files

Supplementary Table 1 **p. 2**

Patients' characteristics

Supplementary Table 2 **p. 3**

Breakdown of patients' characteristic according to FHC grouping across both the cohorts

Supplementary Table 3 **p. 4**

Detailed FHC information for FHC-high patients of the pembrolizumab cohort.

Supplementary Figure 1 **p. 5**

Supplementary Table 4 **p. 6**

Summary the clinical outcomes analysis across the two entire cohorts.

Supplementary Figure 2 **p. 7**

Supplementary Table 5 **p. 8**

Pooled multivariable analysis

Supplementary Figure 3 **p. 10**

Material and Methods **p. 11**

Participating institution list **p. 14**

Material and Methods references **p. 15**

	PEMBROLIZUMAB COHORT 723 N° (%)	CHEMOTHERAPY COHORT 652 N° (%)	
**Age, (years)**			**χ2 test**
Median<br>Range<br>Elderly (≥ 70)	69<br>28 – 92<br>354 (49.0)	68<br>31 – 92<br>283 (43.4)	P = 0.0391
**Gender**			
Female<br>Male	255 (35.3)<br>468 (64.7)	205 (31.4)<br>447 (68.6)	P = 0.1332
**ECOG-PS**			
0 - 1<br>≥ 2	596 (82.4)<br>127 (17.6)	544 (85.1)<br>97 (14.9)	P = 0.1778
**Histology**			
Squamous<br>Non-squamous	174 (24.1)<br>549 (75.9)	140 (21.5)<br>512 (78.5)	P = 0.2527
**Smoking status**			
Never smokers<br>Current/Former smokers	90 (12.4)<br>633 (87.6)	82 (12.6)<br>570 (87.4)	P = 0.1011
**CNS metastases**			
No<br>Yes	589 (81.5)<br>134 (18.5)	544 (83.4)<br>108 (16.6)	P = 0.3385
**Liver metastases**			
No<br>Yes	601 (83.1)<br>122 (16.9)	561 (86.0)<br>91 (14.0)	P = 0.1356
**Bone metastases**			
No<br>Yes	490 (67.8)<br>233 (32.2)	453 (69.5)<br>199 (30.5)	P = 0.4965
**FHC**			
Negative<br>Low<br>High	452 (62.5)<br>222 (30.7)<br>49 (6.8)	389 (59.7)<br>202 (31.0)<br>61 (9.4)	P = 0.1907
**Chemotherapy regimen**			
Platinum-based doublets<br>Single-agent chemotherapy	-	564 (86.5%)<br>88 (13.5%)	-
**Post-progression PD-1/PD-L1 inhibitors**	-	315 (48.3%)	-

Supplementary Table 1: patients' characteristics for both the pembrolizumab and chemotherapy cohorts. ECOG-PS: Eastern Cooperative Oncology Group-Performance Status; CNS: Central Nervous System; FHC: Family History of Cancer.

	PEMBROLIZUMAB COHORT			CHEMOTHERAPY COHORT		
**Age, (years)**	**FHC-high 49 N° (%)**	**FHC-low/negative 674 N° (%)**	**χ2 test**	**FHC-high 61 N° (%)**	**FHC-low/negative 591 N° (%)**	**χ2 test**
Median<br>Range<br>Elderly (≥ 70)	69<br>56 – 80<br>27 (46.9)	69<br>28 – 92<br>331 (49.1)	P = 0.7693	69<br>51 – 84<br>27 (44.3)	68<br>31 – 92<br>256 (43.3)	P = 0.8872
**Gender**						
Female<br>Male	18 (36.7)<br>31 (63.3)	237 (35.2)<br>437 (64.8)	P = 0.8242	17 (27.9)<br>44 (72.1)	188 (31.8)<br>403 (68.2)	P = 0.5282
**ECOG PS**						
0 - 1<br>≥ 2	42 (85.7)<br>7 (14.3)	554 (82.2)<br>120 (17.8)	P = 0.5323	52 (85.2)<br>9 (14.8)	503 (85.1)<br>88 (14.9)	P = 0.9774
**Histology**						
Squamous<br>Non-squamous	15 (30.6)<br>34 (69.4)	159 (23.6)<br>515 (76.4)	P = 0.2673	8 (13.1)<br>53 (86.9)	132 (22.3)<br>459 (77.7)	P = 0.0952
**Smoking status**						
Never smokers<br>Current/Former smokers	4 (8.2)<br>45 (91.8)	86 (12.8)<br>588 (87.2)	P = 0.3470	54 (88.5)<br>7 (11.5)	535 (90.5)<br>56 (9.5)	P = 0.6150
**CNS metastases**						
No<br>Yes	44 (89.8)<br>5 (10.2)	545 (80.9)<br>129 (19.1)	P = 0.1204	49 (80.3)<br>12 (19.7)	495 (83.8)<br>96 (16.2)	P = 0.4932
**Liver metastases**						
No<br>Yes	45 (91.8)<br>4 (8.2)	556 (82.5)<br>118 (17.5)	P = 0.0920	54 (88.5)<br>7 (11.5)	507 (85.8)<br>84 (14.2)	P = 0.5572
**Bone metastases**						
No<br>Yes	36 (73.5)<br>13 (26.5)	454 (67.4)<br>220 (32.6)	P = 0.3772	47 (77.0)<br>14 (23.0)	406 (68.7)<br>185 (31.3)	P = 0.1778
**PD-L1 TPS (536 patients)¥**						
Median value (IQR)	70 (60-80)	0 (60-80)	P = 0.7611	Missing	Missing	-

Supplementary Table 2: breakdown of patients' characteristic according to FHC grouping across both the cohorts. ECOG-PS: Eastern Cooperative Oncology Group-Performance Status; CNS: Central Nervous System; FHC: Family History of Cancer; TPS: tumour proportion score; IQR: interquartile range. ¥ for 187 patients PD-L1 TPS was available as ≥ 50% only.

PATIENT N°	FAMILY HISTORY OF CANCER DESCRIPTIVE
1	**Mother:** lung cancer; **Brother:** lung cancer
2	**Father:** unknown/do not remember; **Sister:** endometrial cancer
3	**Father:** CNS tumor; **Mother:** colon cancer; **Sister:** lung cancer
4	**Mother:** colon cancer; **Sister:** breast cancer
5	**Mother:** leukemia; **Sister:** gastric cancer; **Sister:** lymphoma
6	**Father:** colon cancer; **Sister:** melanoma
7	**Father:** Head and Neck cancer; **Brother**: bladder cancer
8	**Mother:** unknown/do not remember; **Brother:** colon cancer
9	**Mother:** pancreatic cancer; **Sister**: lung cancer
10	**Mother:** endometrial cancer; **Brother:** renal cancer/Thyroid, Lung cancer
11	**Mother**: leukemia; **Sister:** cervical cancer
12	**Father:** lung cancer; **Brother:** melanoma
13	**Mother**: colon cancer; **Sister:** lymphoma
14	**Father:** Head and Neck cancer; **Brother:** colon cancer
15	**Father:** lung cancer; **Brother:** colon cancer
16	**Father:** prostate cancer; **Brother:** lung and Head and Neck cancer
17	**Daughter:** breast cancer; **Sister:** ovarian cancer; **Brother:** melanoma; **Sister:** Head and Neck cancer
18	**Mother:** biliary tract cancer; **Father:** Head and Neck cancer; **Sister:** Thyroid cancer
19	**Mother:** ovarian cancer; **Sister:** ovarian cancer
20	**Grandfather:** GI not specified; **Brother:** unknown/do not remember
21	**Mother:** breast cancer; **Sister:** unknown/do not remember
22	**Grandfather:** Head and Neck cancer; **Sister:** unknown/do not remember
23	**Mother:** breast cancer; **Father:** colon cancer; **Brother:** colon cancer
24	**Mother:** ovarian cancer; **Sister:** breast cancer
25	**Father:** lung cancer; **Brother**: colon cancer
26	**Mother:** Head and Neck cancer; **Grandson:** renal cancer; **Brother:** CNS neoplasm
27	**Grandfather**: lung cancer; **Father**: lung cancer; **Brother**: lung cancer
28	**Grandfather:** lung cancer; **Brother**: unknown/do not remember
29	**Father:** lung cancer; **Mother:** unknown/do not remember; **Sister:** colon cancer
30	**Mother:** breast cancer; **Brother:** unknown/do not remember
31	**Mother:** breast cancer; **Sister:** unknown/do not remember
32	**Father:** lung cancer; **Brother:** lung cancer
33	**Mother:** hepatocellular carcinoma; **Father:** leukemia; **Sister:** ovarian cancer
34	**Father:** lung cancer; **Brother**: lung cancer; **Brother**: hepatocellular carcinoma
35	**Mother:** unknown/do not remember; **Brother**: unknown/do not remember
36	**Father:** colon cancer; **Brother:** unknown/do not remember
37	**Father:** unknown/do not remember; **Daughter:** breast cancer
38	**Father:** lung cancer; **Sister**: leukemia
39	**Mother:** Head and Neck carcinoma; B**rother**: hepatocellular carcinoma
40	**Mother**: colon cancer; **Father:** prostate cancer; **Sister**: breast cancer
41	**Father:** gastric cancer; **Son:** bladder cancer; **Sister:** leukemia
42	**Father:** unknown/do not remember; **Sister:** breast cancer
43	**Son:** penile cancer; **Daughter:** breast cancer; **Brother:** colon cancer; **Brother:** prostate cancer
44	**Father:** Head and Neck cancer; **Brother:** gastric cancer
45	**Mother:** GI cancer; **Son:** unknown/do not remember; **Brother:** unknown/do not remember
46	**Grandfather:** unknown/do not remember; **Son:** lymphoma; **Sister:** unknown/do not remember
47	**Father:** lung cancer; **Brother:** bladder cancer
48	**Mother:** pancreatic cancer; **Brother:** prostate cancer
49	**Father:** Head and Neck cancer; **Sister**: breast cancer

Supplementary Table 3: Detailed FHC information for FHC-high patients of the pembrolizumab cohort.

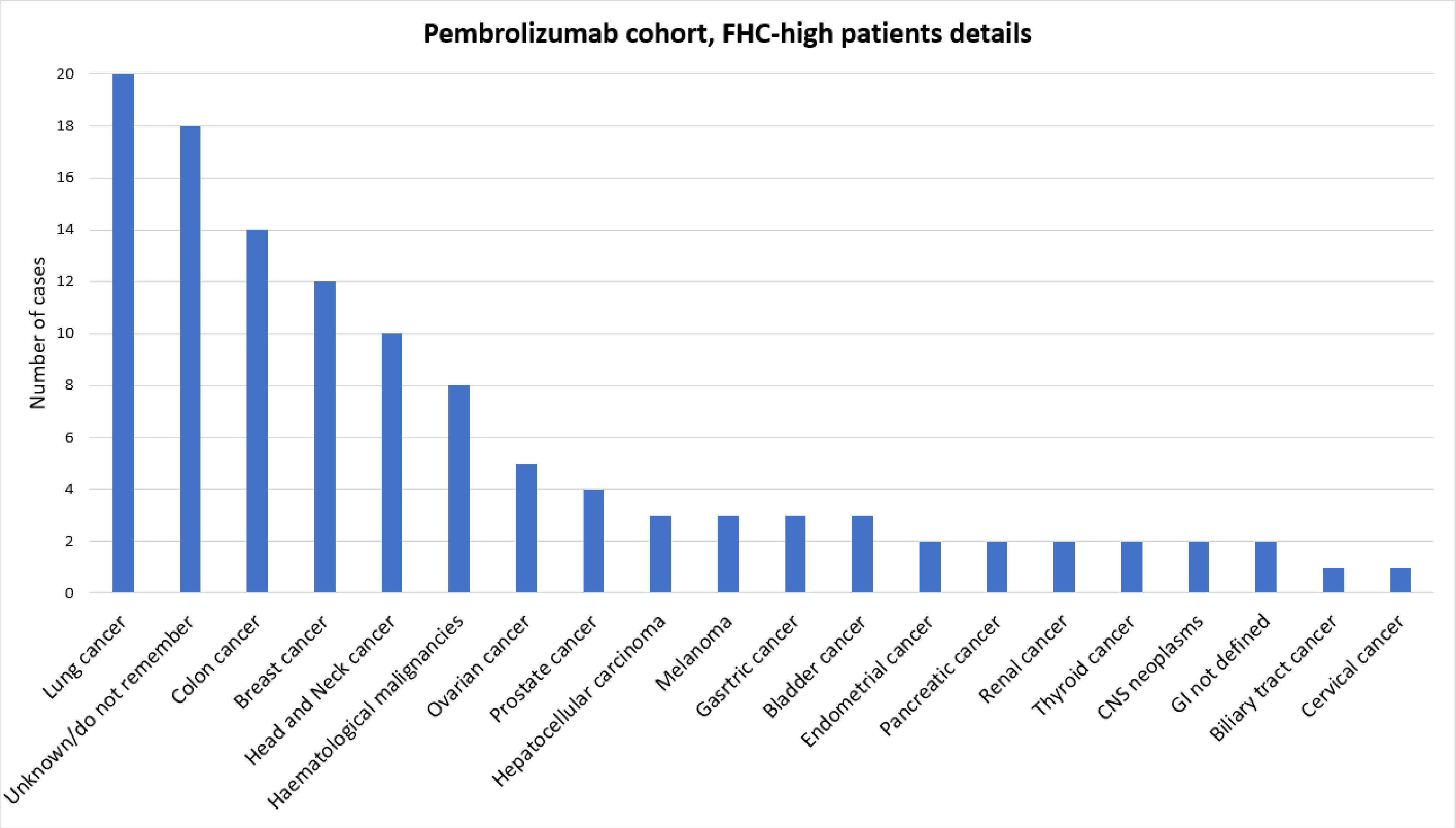

Supplementary Figure 1: Detailed FHC information for FHC-high patients of the pembrolizumab cohort. CNS: Central Nervous System; GI: Gastro-Intestinal.

	PEMBROLIZUMAB COHORT			CHEMOTERAPY COHORT		
**FHC**	**Response/ratio**	**ORR (95%CI)**	**χ2 test**	**Response/ratio**	**ORR (95%CI)**	**χ2 test**
HIGH<br>NON-HIGH	21/46<br>259/613	45.7% (28.2-69.7)<br>42.3% (37.2-47.7)	P = 0.6529	9/41<br>176/477	22.0% (10.0-41.7)<br>36.9% (31.6-42.7)	P = 0.0555
	**Disease control/ratio**	**DCR (95%CI)**	**χ2 test**	**Disease control/ratio**	**DCR (95%CI)**	**χ2 test**
HIGH<br>NON-HIGH	40/46<br>402/613	87.0% (62.1-118.4)<br>65.6% (59.3-72.3)	P = 0.0029	29/41<br>305/477	70.7% (47.4-101.0)<br>63.9% (56.9-71.5)	P = 0.3838
	**PFS (months) (95%CI) [events]**	**log-rank**	**HR (95%CI)**	**PFS (months) (95%CI) [events]**	**log-rank**	**HR (95%CI)**
HIGH<br>NON-HIGH	15.6 (8.6 – 28.2) [31]<br>6.3 (5.4 – 7.5) [492]	P = 0.0100	0.62 (0.43-0.89)	6.0 (4.0 – 48.6) [59]<br>5.9 (5.3 – 6.4) [535]	P = 0.6905	1.05 (0.80-1.38)
	**OS (months) (95%CI) [events]**			**OS (months) (95%CI) [events]**		
HIGH<br>NON-HIGH	31.3 (15.2 – 31.3) [24]<br>14.3 (12.0 – 17.1) [397]	P = 0.0438	0.65 (0.43-0.99)	20.8 (12.7 – 34.5) [38]<br>13.9 (12.6 – 16.2) [428]	P = 0.0307	0.69 (0.49-0.97)

Supplementary Table 4: Summary the clinical outcomes analysis across the two cohorts. ECOG-PS: Eastern Cooperative Oncology Group-Performance Status; CNS: Central Nervous System; FHC: Family History of Cancer; ORR: Objective Response Rate; DCR: Disease Control Rate; PFS; Progression Free Survival; OS: Overall Survival; HR: Hazard Ratio; CI: Confidence Interval. Only 659 and 518 patients were evaluable for ORR and DCR in the pembrolizumab and chemotherapy cohort respectively.

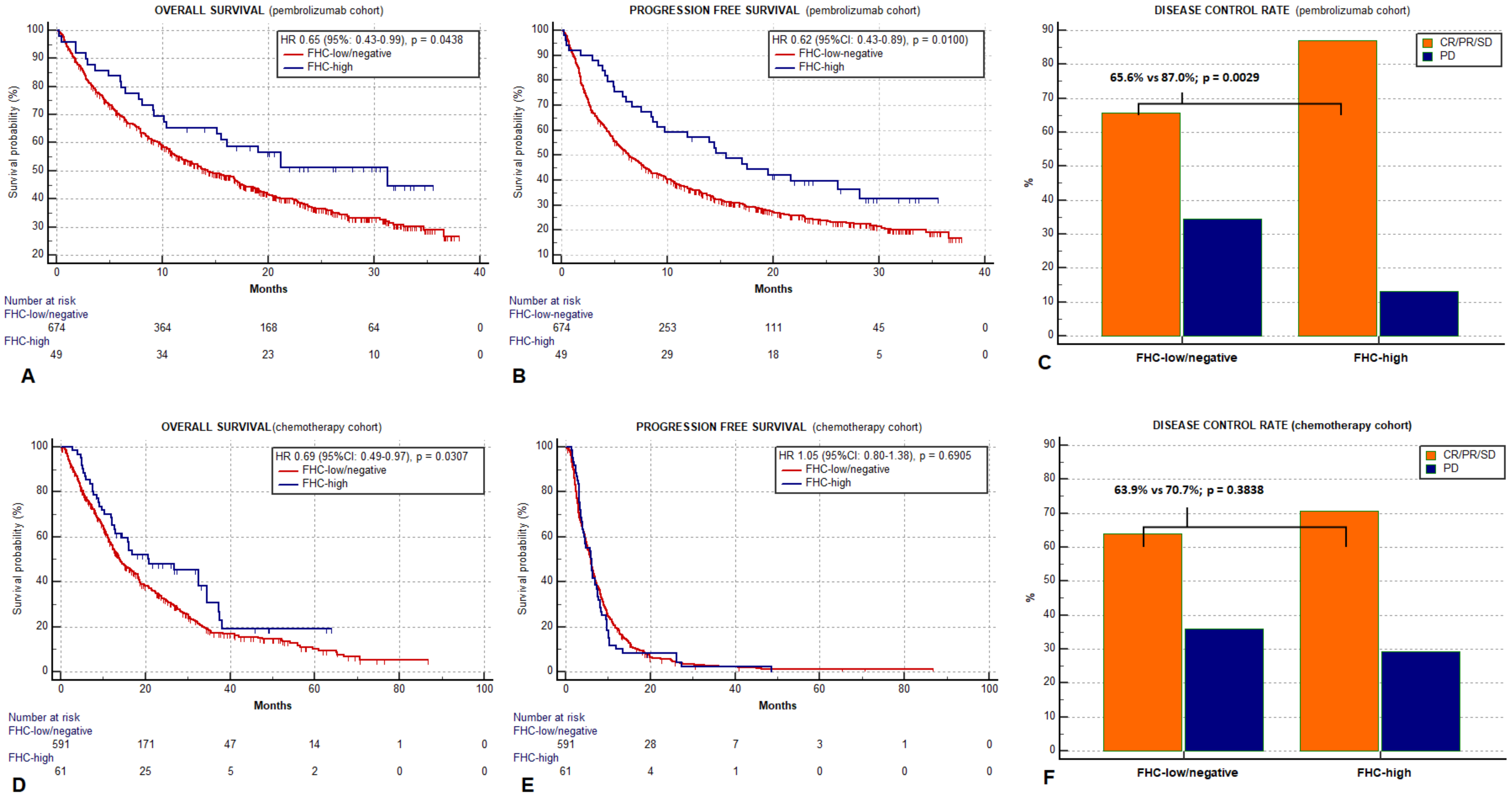

Supplementary Figure 2: Clinical outcomes analysis according to the FHC the pembrolizumab and chemotherapy entire cohorts (also reported in **Supplementary Table 4**). (**A**) Pembrolizumab cohort, Kaplan-Meier survival estimate for Overall Survival. (**B**) Pembrolizumab cohort, Kaplan-Meier survival estimate for Progression Free Survival. (**C**) Pembrolizumab cohort, Frequency chart for disease control rate. (**D**) Chemotherapy cohort, Kaplan-Meier survival estimate for Overall Survival. (**E**) Chemotherapy cohort, Kaplan-Meier survival estimate for Progression Free Survival. (**F**) Chemotherapy cohort, Frequency chart for disease control rate.

	POOLED ANALYSIS (without interaction)		
	DISEASE CONTROL RATE	PROGRESSION FREE SURVIVAL	OVERALL SURVIVAL
**VARIABLE**	**OR (95% CI);** ***p - value***	**HR (95% CI);** ***p - value***	**HR (95% CI);** ***p - value***
**Cohort**<br>Pembrolizumab vs Chemotherapy	1.16 (0.90-1.50); p = 0.2298	0.61 (0.54-0.69); p < 0.0001	0.91 (0.79-1.04); p = 0.1876
**FHC**<br>High vs Non-high	1.88 (1.09-3.27); p = 0.0233	0.87 (0.70-1.08); p = 0.2306	0.67 (0.51-0.87); p = 0.0028
**Gender**<br>Male vs Female	0.95 (0.72-1.26); p = 0.7373	1.13 (0.99-1.29); p = 0.0687	1.13 (0.97-1.31); p = 0.1005
**Age**<br>Elderly vs Non-elderly	0.94 (0.72-1.21); p = 0.6444	1.08 (0.96-1.22); p = 0.1775	1.25 (1.09-1.43); p = 0.0011
**ECOG PS**<br>≥2 vs 0-1	0.34 (0.24-0.47); p < 0.0001	1.95 (1.66-2.28); p < 0.0001	2.44 (2.06-2.89); p < 0.0001
**Smoking status**<br>Never vs Current/former	0.72 (0.48-1.08); p = 0.1198	1.31 (1.09-1.58); p = 0.0040	1.07 (0.86-1.33); p = 0.4912
**CNS metastases**<br>Yes vs No	1.20 (0.84-1.70); p = 0.3125	1.11 (0.94-1.30); p = 0.1877	1.22 (1.03-1.46); p = 0.0208
**Bone metastates**<br>Yes vs No	0.58 (0.44-0.75); p = 0.0001	1.42 (1.25-1.61); p < 0.0001	1.41 (1.22-1.62); p < 0.0001
**Liver metastases**<br>Yes vs No	0.52 (0.37-0.72); p = 0.0001	1.56 (1.33-1.83); p < 0.0001	1.38 (1.16-1.65); p = 0.0003
**Chi-squared statistic for the overall model fit**	94.4, DF: 9; p < 0.0001	210.6, DF 9; p < 0.0001	176.1, DF 9; p < 0.0001
	**POOLED ANALYSIS (with interaction)**		
**Cohort**<br>Pembrolizumab vs Chemotherapy	1.10 (0.85-1.43); p = 0.4413	0.63 (0.56-0.72); p < 0.0001	0.90 (0.79-1.04); p = 0.1798
**FHC**<br>High vs Non-high	1.23 (0.60-2.52); p = 0.5707	1.09 (0.83-1.43); p = 0.4937	0.65 (0.46-0.91); p = 0.0129
**Interaction**<br>FHC*Cohort	**p = 0.1020**	**p = 0.0170**	**p = 0.7923**
**Gender**<br>Male vs Female	0.95 (0.72-1.26); p = 0.7646	1.12 (0.98-1.28); p = 0.0864	1.13 (0.97-1.31); p = 0.0985
**Age**<br>Elderly vs Non-elderly	0.94 (0.73-1.22); p = 0.6648	1.09 (0.96-1.23); p = 0.1597	1.25 (1.09-1.43); p = 0.0011
**ECOG PS**<br>≥2 vs 0-1	0.33 (0.24-0.47); p < 0.0001	1.95 (1.67-2.28); p < 0.0001	2.45 (2.07-2.89); p < 0.0001
**Smoking status**<br>Never vs Current/former	0.73 (0.49-1.10); p = 0.1406	1.30 (1.08-1.57); p = 0.0047	1.07 (0.86-1.34); p = 0.4896
**CNS metastases**	1.21 (0.85-1.72); p = 0.2843	1.10 (0.94-1.29); p = 0.2243	1.22 (1.03-1.46); p = 0.0206

Yes vs No			
**Bone metastates** Yes vs No	0.57 (0.44-0.75); p < 0.0001	1.43 (1.25-1.62); p < 0.0001	1.41 (1.22-1.62); p < 0.0001
**Liver metastases** Yes vs No	0.52 (0.37-0.73); p = 0.0002	1.55 (1.33-1.82); p < 0.0001	1.38 (1.16-1.65); p = 0.0003
**Chi-squared statistic for the overall model fit**	97.2, DF: 10; p < 0.0001	216.6, DF 10; p < 0.0001	176.1, DF 10; p < 0.0001

Supplementary Table 5: Summary of the pooled multivariable analysis for DCR, PFS and OS within the pembrolizumab cohort without and with the interaction term FHC*Cohort. ECOG-PS: Eastern Cooperative Oncology Group-Performance Status; CNS: Central Nervous System; FHC: Family History of Cancer; DCR: Disease Control Rate; PFS; Progression Free Survival; OS: Overall Survival; OR: Odd Ratio; HR: Hazard Ratio; CI: Confidence Interval.

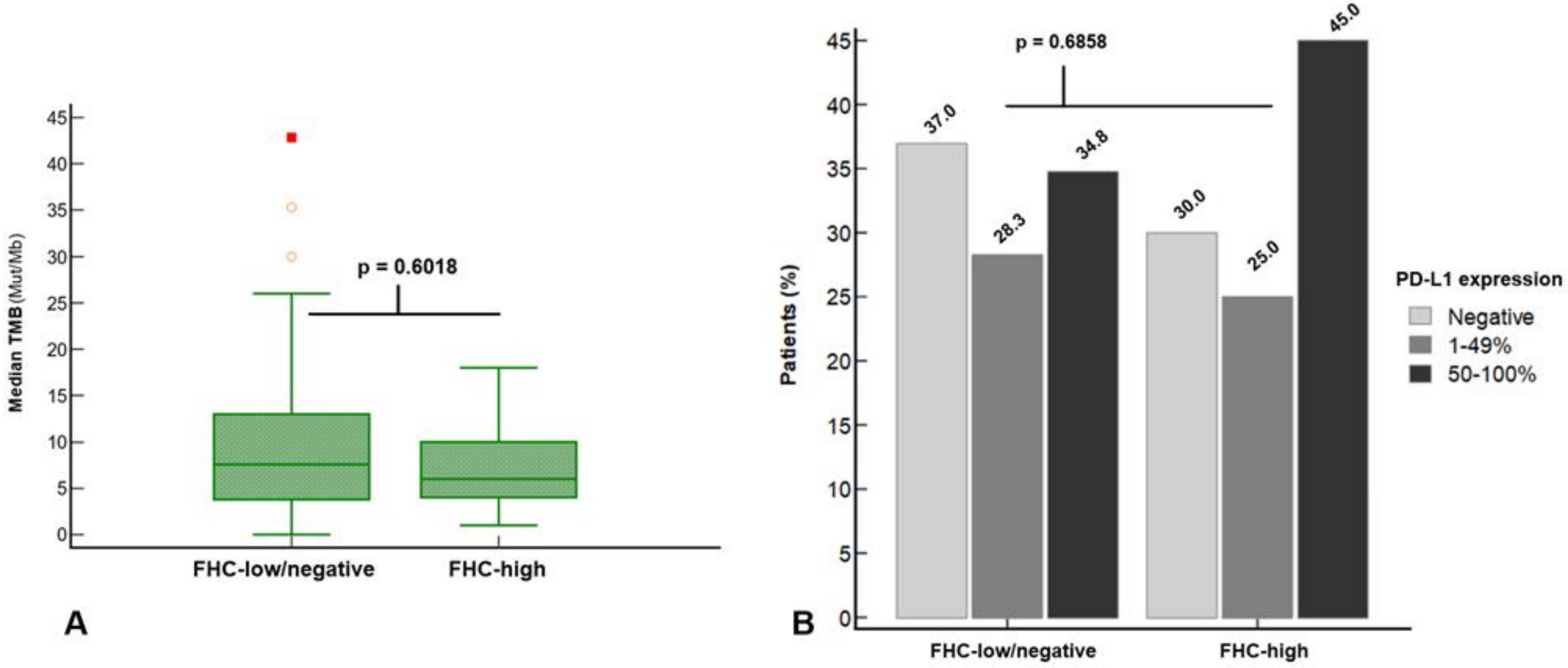

Supplementary Figure 3: FDx cohort (**A**) Multiple comparison graph for the median TMB according to the FHC grouping: the median TMB for FHC-high was 6 Mut/Mb (range: 1–18), whilst for the FHC-low/negative was 7.6 Mut/Mb (range: 0–42.8) (p=0.6018). (**B**) Frequency chart for the PD-L1 expression distribution according to the FHC grouping. Frequencies are reported in %. TMB: umour mutational burden.

Materials and Methods

Study design

The main aim of this study was to evaluate the role of FHC in a cohort of patients with metastatic NSCLC with a PD-L1 tumour expression ≥ 50%, treated with first-line pembrolizumab monotherapy [1-8]. Following a data request update, 29 institutions participated to the study and retrospectively included patients treated from January 2017 to May 2020.

In order to assess the potential different impact of FHC depending on the treatment strategy, we evaluated a second cohort of patients with metastatic *EGFR* (Epidermal Growth Factor Receptor) wild type NSCLC (ALK and ROS-1 unknown) treated with first line chemotherapy at 13 of the participating institutions from January 2013 to May 2020; the censoring date was 30 September 2020.

Study endpoints included objective response rate (ORR), disease control rate (DCR), PFS and OS. Patients were assessed with radiological imaging at participating institutions, with a frequency ranging from 8 to 12 weeks; investigator-assessed disease response followed Response Evaluation Criteria in Solid Tumors (RECIST) criteria v1.1. PFS and OS were measured from treatment initiation to disease progression and/or death. Patients without documented disease progression at the data cut off were censored on the date of last clinical follow-up and radiological assessment for OS and PFS, respectively.

PD-L1 expression analysis among the entire population has been already reported [1]. Considering that tumour proportion score (TPS) for PD-L1 expression has been validated with the 22C3 antibody only, we referred to "PD-L1 expression" throughout all the study. All the immunohistochemical (IHC) analyses were preformed locally at each participating institution, using a different antibodies and platforms according to their respective clinical practice (including 22C3 [60.4%], SP263 [32.1%], E1L3N [0.9%], 28-8 [1.7%], not available [4.9%]). Considering that in some institutions the PD-L1 expression level is reported only as "≥ 50%", and not as a discrete value, only patients with data availability regarding the absolute value of PD-L1 tumour staining have been included in the association analysis between PD-L1 expression and FHC.

To estimate the differential impact of the FHC across the two populations, we evaluated the impact of FHC on clinical outcomes after a perfect random case-control matching between the two cohorts. Cases and controls were randomly paired on the basis of the FHC, age (< 70 vs. ≥ 70 years old), ECOG-PS (0-1 vs ≥ 2), and burden of disease (≥ 2 vs < two metastatic sites).

We then explored the impact of the FHC within the pembrolizumab and chemotherapy cohorts using univariable analyses. A fixed regression model including major determinants of clinical outcome within the study population [1-8] was used for the multivariable analysis of the pembrolizumab cohort. Additionally, to further evaluate the role of FHC depending on the treatment modality (immunotherapy vs chemotherapy), we performed a pooled analysis of both the cohorts, with and without the interaction term between the FHC and the therapeutic modality (pembrolizumab vs chemotherapy).

Definition of family history of cancer

Family history data was collected from medical records as previously described, and all oncological disease with malignant potential, both hematological and solid, were screened [9]. Lineal line (descendants or ascendants) and collateral line (non-descentants/ascendants e.g., brothers/sisters) were screened till the second degree (grandparents for lineal line and brothers/sisters for the collateral line). Patients were categorized as follow: FHC-high (in case of at least one cancer diagnosis in both lineal and collateral family lines), FHC-low (in case of at least one cancer diagnosis in either the lineal or collateral line) and FHC-negative (**Figure 1**). On the basis of our previous findings [9], FHC-high was considered the group of interest for all analyses.

DDR genes exploratory analysis - FDx cohort.

We used a parallel cohort of patients with NSCLC from 4 of the participating institutions (reported in **Supplementary Table 1**), in order to explore the putative role of DDR genes alterations as underlying mechanism to the effect of FHC on clinical outcomes. Relevant baseline clinico-pathologic data and tumour genomic information were gathered. The targeted DNA tumour sequencing was performed with the FoundationOne CDx assay (Detailed information on the variant calling and functional evaluation are available at https://info.foundationmedicine.com/hubfs/FMI%20Labels/FoundationOne_CDx_Label_Technical_Info.pdf).

The panel of 53 genes related to DNA damage response and repair defined by Ricciuti et al. in a large cohort of patients with NSCLC [10] was used as reference, to verify their possible association with FHC. We identified 24 genes of interest among the 324 cancer-related genes and selected rearrangement detected by the FoundationOne CDx assay (1. mismatch repair: *MLH1*, *MSH6*, *PMS2;* 2. DNA damage sensing: *ATM*, *ATR*, *CHEK1*, *CHEK2;* 3. homologous recombination *BAP1*, *BARD1*, *BRCA1*, *BRCA2*, *BRIP1*, *PALB2*, *RAD51*, *RAD51C*, *RAD52;* 4. Fanconi anemia: *FANCA*, *FANCC*, *FANCG*, *FANCL;* 5. DNA polymerase: *POLD1*, *POLE;* 6. nucleotide excision repair: *ERCC4;* 7. base excision repair: *XRCC2*). We also explored the associations between FHC, median tumor mutational burden (TMB), smoking status and PD-L1 tumour expression.

Statistical Analysis

The sample size was estimated for the pembrolizumab cohort only, on the basis of the expected number of FHC high patients. According to the subgroup analysis on NSCLC patients evaluated within our previous study [9], we hypothesized a 11% prevalence of FHC high patients and assumed a survival benefit for FHC high patients compared to non-FHC high, with a reduction of the risk of death by 56%. With a probability of Type I error of 0.05 and of Type II error of 0.20, 238 total events were necessary and at least 633 patients had to be recruited overall from the original cohort. Baseline patients'

characteristics were reported with summative descriptive statistics (means, medians and proportions) as appropriate. $\chi 2$ test and Fisher's exact test were used to compare categorical variables as appropriate. The Kruskal-Wallis test was used to compare median TMB according to the FHC and to evaluate the associations between FHC and PD-L1 tumor expression among the pembrolizumab cohort. Median PFS and median OS were evaluated using the Kaplan-Meier method and the log-rank test. Median period of follow-up was calculated according to the reverse Kaplan-Meier method. Logistic regression was used for the multivariable analysis of DCR and to compute odds radios (OR) with 95% confidence intervals (Cis). Cox proportional hazards regression was used for the multivariable analysis of PFS and OS and to compute all the hazard ratios (HR) for disease progression and death with 95% CIs. Considering that all the selected variables were categorical, a caliper width of < 1 for the standard deviation was used for the random case-control matching. The alpha level for all analyses was set to $p<0.05$. All statistical analyses were performed using MedCalc Statistical Software version 19.3.1 (MedCalc Software Ltd, Ostend, Belgium; https://www.medcalc.org; 2020).

**Institution**
St. Salvatore Hospital, University of L’Aquila, L’Aquila *
SS Annunziata Hospital, Chieti *
University Hospital of Parma, Parma
St. Camillo Forlanini Hospital, Rome
University Hospital of Modena, Modena
S Maria Goretti Hospital, Latina *
St. Andrea Hospital, Rome * ¥
Fondazione IRCCS Ca' Granda Ospedale Maggiore Policlinico, Milan *
Campus Bio-Medico University, Rome * ¥
AOU Papardo, Messina * ¥
“Ospedali Riuniti” Hospital, Ancona *
Policlinico Umberto I, Rome
Azienda Ospedaliera Santa Maria, Terni
AUSL Latina, Aprilia *
“Augusto Murri” Hospital, Fermo
IRCCS – Istituto Nazionale Tumori, Fondazione “G. Pascale”, Napoli
IRCCS Sacro Cuore Don Calabria, Negrar
University Hospital of Udine, Udine
ASST-Sette Laghi, Varese *
University Hospital “A.Gemelli”, Rome
“Madre Teresa Di Calcutta” Hospital Padova Sud, Monselice
“F. Spaziani” Hospital, Frosinone
“Careggi” University Hospital, Florence ¥
“Monaldi” Hospital, Naples *
Erasmus Medical Center, Rotterdam, the Netherlands
Imperial College London, United Kingdome
“Santa Maria della Misericordia” Hospital, Perugia *
University Hospital of Geneva, Geneva *
IRCCS, Policlinico San Martino, Genova

List of the participating Institutions. * Intitutions which provided data for the chemotherapy cohort. ¥ Institution which provided data for the FDX cohort.

Material and Methods references:

1. Cortellini A, Tiseo M, Banna GL, et al. Clinicopathologic correlates of first-line pembrolizumab effectiveness in patients with advanced NSCLC and a PD-L1 expression of ≥ 50% Cancer Immunol Immunother. 2020 Nov;69(11):2209-2221. doi: 10.1007/s00262-020-02613-9. Epub 2020 May 30.

2. Cortellini A, Friedlaender A, Banna GL, et al. Immune-related Adverse Events of Pembrolizumab in a Large Real-world Cohort of Patients With NSCLC With a PD-L1 Expression ≥ 50% and Their Relationship With Clinical Outcomes Clin Lung Cancer. 2020 Jun 21:S1525-7304(20)30204-7. doi: 10.1016/j.cllc.2020.06.010. Epub ahead of print.

3. Cortellini A, Ricciuti B, Tiseo M, et al. Baseline BMI and BMI variation during first line pembrolizumab in NSCLC patients with a PD-L1 expression ≥ 50%: a multicenter study with external validation. J Immunother Cancer. 2020 Oct;8(2):e001403. doi: 10.1136/jitc-2020-001403.

4. Banna GL, Cortellini A, Cortinovis DL, et al. The lung immuno-oncology prognostic score (LIPS-3): a prognostic classification of patients receiving first-line pembrolizumab for PD-L1 ≥ 50% advanced non-small-cell lung cancer. ESMO Open. 2021 Apr;6(2):100078. doi: 10.1016/j.esmoop.2021.100078. Epub 2021 Mar 16.

5. Cortellini A, De Giglio A, Cannita K, et al. Smoking status during first-line immunotherapy and chemotherapy in NSCLC patients: A case-control matched analysis from a large multicenter study. Thorac Cancer. 2021 Mar;12(6):880-889. doi: 10.1111/1759-7714.13852. Epub 2021 Feb 1.

6. Cortellini A, Cannita K, Tiseo M, et al. Post-progression outcomes of NSCLC patients with PD-L1 expression ≥ 50% receiving first-line single-agent pembrolizumab in a large multicentre real-world study. Eur J Cancer. 2021 May;148:24-35. doi: 10.1016/j.ejca.2021.02.005. Epub 2021 Mar 12.

7. Cortellini A, Di Maio M, Nigro O, et al. Differential influence of antibiotic therapy and other medications on oncological outcomes of patients with non-small cell lung cancer treated with first-line pembrolizumab versus cytotoxic chemotherapy. J Immunother Cancer. 2021 Apr;9(4):e002421. doi: 10.1136/jitc-2021-002421.

8. Buti S, Bersanelli M, Perrone F, et al. Predictive ability of a drug-based score in patients with advanced non-small-cell lung cancer receiving first-line immunotherapy. Eur J Cancer. 2021 Jun;150:224-231. doi: 10.1016/j.ejca.2021.03.041. Epub 2021 May 3.

9. Cortellini A, Buti S, Bersanelli M, et al. Evaluating the role of FAMIly history of cancer and diagnosis of multiple neoplasms in cancer patients receiving PD-1/PD-L1 checkpoint inhibitors: the multicenter FAMI-L1 study. Oncoimmunology. 2020 Jan 7;9(1):1710389. doi: 10.1080/2162402X.2019.1710389.

10. Ricciuti B, Recondo G, Spurr LF, et al. Impact of DNA Damage Response and Repair (DDR) Gene Mutations on Efficacy of PD-(L)1 Immune Checkpoint Inhibition in Non-Small Cell Lung Cancer. Clin Cancer Res. 2020 Aug 1;26(15):4135-4142. doi: 10.1158/1078-0432.CCR-19-3529. Epub 2020 Apr 2416.
